# 1′-Ethyl­sulfanyl-1,1′-bicyclo­hexyl-2-one

**DOI:** 10.1107/S1600536810010731

**Published:** 2010-03-27

**Authors:** Lalit Kumar Sharma, Sean Parkin, Gregory I. Elliott

**Affiliations:** a Department of Chemistry, University of Kentucky, Lexington, KY 40506, USA; b Department of Pharmaceutical Sciences, College of Pharmacy, University of Kentucky, Lexington, KY 40536, USA

## Abstract

There are two independent molecules in the asymmetric unit of the title cyclo­hexa­none derivative, C_14_H_24_OS, in which both cyclo­hexane rings exhibit chair conformations. They are also equatorial to each other, which permits the ethanethiol substituent to be in a *syn* conformation with the α-H atom of the parent attached cyclo­hexa­none.

## Related literature

For background literature on the synthesis, see Bach & Klix (1985 ▶); Trost *et al.* (1976 ▶); Reetz & Giannis (1981 ▶). For the preparation of the starting materials, see: Ito *et al.* (1979 ▶); Kumar & Dev (1983 ▶).
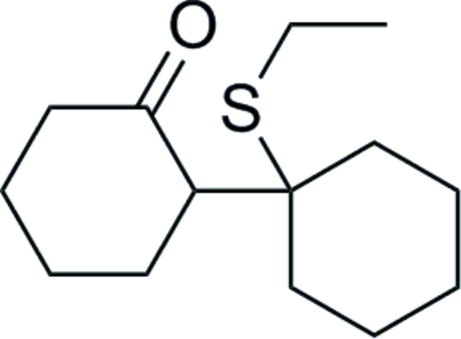


## Experimental

### 

#### Crystal data

C_14_H_24_OS
*M* *_r_* = 240.39
Triclinic, 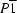

*a* = 10.3662 (2) Å
*b* = 11.2090 (2) Å
*c* = 11.5026 (2) Å
α = 92.5786 (8)°
β = 101.7513 (8)°
γ = 90.2145 (8)°
*V* = 1307.09 (4) Å^3^
*Z* = 4
Mo *K*α radiation
μ = 0.23 mm^−1^
*T* = 90 K
0.18 × 0.15 × 0.10 mm

#### Data collection

Nonius KappaCCD diffractometer
Absorption correction: multi-scan (*SCALEPACK*; Otwinowski & Minor, 1997 ▶) *T*_min_ = 0.960, *T*_max_ = 0.978
32350 measured reflections
5986 independent reflections
5000 reflections with *I* > 2σ(*I*)
*R*_int_ = 0.037

#### Refinement

*R*[*F* ^2^ > 2σ(*F* ^2^)] = 0.034
*wR*(*F* ^2^) = 0.092
*S* = 1.05
5986 reflections
291 parameters
H-atom parameters constrained
Δρ_max_ = 0.40 e Å^−3^
Δρ_min_ = −0.34 e Å^−3^

### 

Data collection: *COLLECT* (Nonius, 1998 ▶); cell refinement: *SCALEPACK* (Otwinowski & Minor, 1997 ▶); data reduction: *DENZO-SMN* (Otwinowski & Minor, 1997 ▶); program(s) used to solve structure: *SHELXS97* (Sheldrick, 2008 ▶); program(s) used to refine structure: *SHELXL97* (Sheldrick, 2008 ▶); molecular graphics: *XP* in *SHELXTL* (Sheldrick, 2008 ▶); software used to prepare material for publication: *SHELXL97*and local procedures.

## Supplementary Material

Crystal structure: contains datablocks global, I. DOI: 10.1107/S1600536810010731/ng2746sup1.cif

Structure factors: contains datablocks I. DOI: 10.1107/S1600536810010731/ng2746Isup2.hkl

Additional supplementary materials:  crystallographic information; 3D view; checkCIF report

**Fig. 1.**
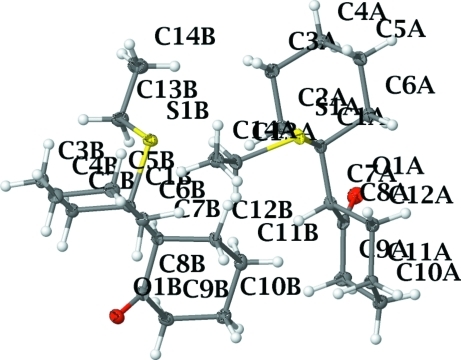
Displacemt ellipsoids drawn at 50% probability level.

Enhanced figure: interactive version of Fig. 1

## supplementary crystallographic information

### Comment

Self condensation of cyclohexanone followed by *in situ* dehydration provides an isomeric mixture of products, specifically, 2-(1-cyclohexen-l-yl)cyclohexanone and cyclohexylidenecyclohexanone. In our efforts to obtain only cyclohexylidinecyclohexanone, an improved route was developed. The title compound, C_14_H_24_OS, was prepared through a Lewis acid mediated alkylation between 1,1-bis(ethylsulfanyl)cyclohexane and 1-trimethylsilyloxycyclohexene at low temperature. The title compound can be oxidized with NaIO_4_ and the corresponding cyclohexylidinecyclohexanone is produced as the only product in moderate yield.

The conformational energy for a cyclohexyl ring is 2.15 kcal/mol while the energy for ethanethiol is approxiamately 0.7 kcal/mol. From this information it is expected that both ring systems would be in an equatorial position leaving the thiol axial. The xray analysis provided agreement to our hypothesis.

### Experimental

SnCl_4 _(10 ml, 1M in CH_2_Cl_2_, 10 mmol) was added to 20 ml of anhydrous CH_2_Cl_2_ at –60°C. A cooled (–60°C) solution of 1,1-bis(ethylsulfanyl)cyclohexane (2.04 g, 10 mmol) in 5 ml anhydrous CH_2_Cl_2_ was added dropwise. Immediately following the final addition of 1,1-bis(ethylsulfanyl)cyclohexane was slowly added a cooled (–60°C) solution of 1-trimethylsilyloxycyclohexene (1.70 g , 10 mmol) in 5 ml anhydrous CH_2_Cl_2_. The solution stirred at –60°C for 45 min and was poured on to 100 ml of ice water. The aqueous phase was extracted with CH_2_Cl_2_(3 X 50 ml). The combined organic phases were washed with 10% aqueous NaHCO_3_ (1 X 100 ml), water (1 X 100 ml) and dried over MgSO_4_. The filtrate was concentrated under reduced pressure providing the title compound (2.05 g) as a white solid. Recrystallization from a solution of hexane–CH_2_Cl_2_ (3:1) provided 1.90 g (79% yield) of 1'-(ethylsulfanyl)-1,1'-bi(cyclohexyl)-2-one. mp = 72°C. ^1^H NMR (CDCl_3_, 500 MHz) *δ* : 2.58–2.52 (m,1*H*); 2.47 (dd, *J*=11.7, 5 Hz, 1H); 2.37 (dq, *J*=7.3, 1 Hz, 2H); 2.34–2.24 (m, 2H); 2.06–1.88 (m, 4H); 1.84–1.74 (m, 1H); 1.74–1.56 (m, 6H); 1.56–1.48 (m, 1H); 1.48–1.40 (m, 2H); 1.32–1.22 (m, 1H); 1.22–1.16 (dt, *J*=7.5, 1.5 Hz, 3H). ^13^C NMR (CDCl_3_, 125 MHz) *δ*: 212.2, 58.5, 52.2, 44.3, 32.6, 31.4, 29.9, 28.6, 25.9, 25.7, 22.0, 21.9, 21.1, 14.1. IR (ν_max_): 2931, 1697, 1500, 1310, 1115, 1063, 884 cm^-1^.

1,1-bis(ethylsulfanyl)cyclohexane was prepared by following the procedure of Kumar & Dev (1983) and 1-trimethylsilyloxycyclohexene was prepared by following the procedure of Ito *et al.* (1979).

### Refinement

H atoms were found in difference Fourier maps and subsequently placed in idealized positions with constrained distances of 0.98 Å (RCH_3_), 0.99 Å (*R*_2_CH_2_), 1.00 Å (*R*_3_CH), and with *U*_iso_(H) values set to either 1.2*U*_eq_ or 1.5*U*_eq_ (RCH_3_) of the attached atom.

### Crystal data

**Table:**

C_14_H_24_OS	*Z* = 4
*M**_r_* = 240.39	*F*(000) = 528
Triclinic, *P*1	*D*_x_ = 1.222 Mg m^−^^3^
Hall symbol: -P 1	Mo *K*α radiation, λ = 0.71073 Å
*a* = 10.3662 (2) Å	Cell parameters from 5942 reflections
*b* = 11.2090 (2) Å	θ = 1.0–27.4°
*c* = 11.5026 (2) Å	µ = 0.23 mm^−^^1^
α = 92.5786 (8)°	*T* = 90 K
β = 101.7513 (8)°	Rod, colourless
γ = 90.2145 (8)°	0.18 × 0.15 × 0.10 mm
*V* = 1307.09 (4) Å^3^	

### Data collection

**Table:**

Nonius KappaCCD diffractometer	5986 independent reflections
Radiation source: fine-focus sealed tube	5000 reflections with *I* > 2σ(*I*)
graphite	*R*_int_ = 0.037
Detector resolution: 9.1 pixels mm^-1^	θ_max_ = 27.5°, θ_min_ = 1.8°
ω scans at fixed χ = 55°	*h* = −13→13
Absorption correction: multi-scan (*SCALEPACK*; Otwinowski & Minor, 1997)	*k* = −14→14
*T*_min_ = 0.960, *T*_max_ = 0.978	*l* = −14→14
32350 measured reflections	

### Refinement

**Table:**

Refinement on *F*^2^	Primary atom site location: structure-invariant direct methods
Least-squares matrix: full	Secondary atom site location: difference Fourier map
*R*[*F*^2^ > 2σ(*F*^2^)] = 0.034	Hydrogen site location: inferred from neighbouring sites
*wR*(*F*^2^) = 0.092	H-atom parameters constrained
*S* = 1.05	*w* = 1/[σ^2^(*F*_o_^2^) + (0.0458*P*)^2^ + 0.4279*P*] where *P* = (*F*_o_^2^ + 2*F*_c_^2^)/3
5986 reflections	(Δ/σ)_max_ = 0.002
291 parameters	Δρ_max_ = 0.40 e Å^−^^3^
0 restraints	Δρ_min_ = −0.34 e Å^−^^3^

### Special details

**Table:**

Geometry. All esds (except the esd in the dihedral angle between two l.s. planes) are estimated using the full covariance matrix. The cell esds are taken into account individually in the estimation of esds in distances, angles and torsion angles; correlations between esds in cell parameters are only used when they are defined by crystal symmetry. An approximate (isotropic) treatment of cell esds is used for estimating esds involving l.s. planes.
Refinement. Refinement of *F*^2^ against ALL reflections. The weighted *R*-factor wR and goodness of fit *S* are based on *F*^2^, conventional *R*-factors *R* are based on *F*, with *F* set to zero for negative *F*^2^. The threshold expression of *F*^2^ > 2σ(*F*^2^) is used only for calculating *R*-factors(gt) etc. and is not relevant to the choice of reflections for refinement. *R*-factors based on *F*^2^ are statistically about twice as large as those based on *F*, and *R*- factors based on ALL data will be even larger.

### Fractional atomic coordinates and isotropic or equivalent isotropic displacement parameters (Å^2^)

**Table:**

	*x*	*y*	*z*	*U*_iso_*/*U*_eq_	
S1A	0.88224 (3)	0.94850 (3)	0.30582 (3)	0.01526 (9)	
O1A	0.53397 (9)	0.80151 (9)	−0.01038 (8)	0.0194 (2)	
C1A	0.72797 (12)	0.89244 (11)	0.20595 (11)	0.0130 (2)	
C2A	0.68219 (13)	0.77443 (11)	0.24908 (11)	0.0152 (3)	
H2A1	0.6038	0.7436	0.1911	0.018*	
H2A2	0.7529	0.7150	0.2511	0.018*	
C3A	0.64777 (14)	0.78678 (12)	0.37204 (12)	0.0182 (3)	
H3A1	0.7281	0.8086	0.4321	0.022*	
H3A2	0.6142	0.7092	0.3924	0.022*	
C4A	0.54321 (14)	0.88253 (12)	0.37498 (12)	0.0195 (3)	
H4A1	0.4601	0.8572	0.3203	0.023*	
H4A2	0.5255	0.8920	0.4563	0.023*	
C5A	0.59008 (13)	1.00195 (12)	0.33805 (12)	0.0172 (3)	
H5A1	0.6682	1.0309	0.3974	0.021*	
H5A2	0.5196	1.0616	0.3367	0.021*	
C6A	0.62570 (13)	0.99037 (12)	0.21515 (11)	0.0154 (3)	
H6A1	0.6612	1.0679	0.1970	0.019*	
H6A2	0.5446	0.9720	0.1547	0.019*	
C7A	0.75555 (12)	0.87558 (11)	0.07876 (11)	0.0132 (2)	
H7A	0.8342	0.8230	0.0853	0.016*	
C8A	0.64834 (13)	0.81697 (11)	−0.01800 (11)	0.0153 (3)	
C9A	0.69592 (14)	0.78561 (12)	−0.13183 (12)	0.0188 (3)	
H9A1	0.6224	0.7492	−0.1917	0.023*	
H9A2	0.7671	0.7263	−0.1158	0.023*	
C10A	0.74760 (13)	0.89752 (12)	−0.18129 (12)	0.0176 (3)	
H10A	0.7887	0.8737	−0.2492	0.021*	
H10B	0.6731	0.9506	−0.2105	0.021*	
C11A	0.84846 (13)	0.96463 (12)	−0.08583 (11)	0.0162 (3)	
H11A	0.8741	1.0401	−0.1171	0.019*	
H11B	0.9284	0.9157	−0.0647	0.019*	
C12A	0.79214 (13)	0.99232 (11)	0.02493 (12)	0.0155 (3)	
H12A	0.7127	1.0419	0.0039	0.019*	
H12B	0.8580	1.0384	0.0847	0.019*	
C13A	0.99018 (13)	0.82077 (12)	0.31977 (12)	0.0166 (3)	
H13A	0.9536	0.7569	0.3608	0.020*	
H13B	0.9986	0.7887	0.2401	0.020*	
C14A	1.12463 (13)	0.86198 (13)	0.39135 (12)	0.0199 (3)	
H14A	1.1623	0.9216	0.3477	0.030*	
H14B	1.1834	0.7934	0.4040	0.030*	
H14C	1.1145	0.8974	0.4684	0.030*	
S1B	0.75895 (3)	0.44991 (3)	0.30868 (3)	0.01604 (9)	
O1B	0.96037 (9)	0.30177 (9)	−0.00856 (8)	0.0195 (2)	
C1B	0.86605 (12)	0.39287 (11)	0.20787 (11)	0.0134 (2)	
C2B	0.92957 (13)	0.27433 (11)	0.25006 (11)	0.0155 (3)	
H2B1	0.8590	0.2149	0.2513	0.019*	
H2B2	0.9818	0.2439	0.1922	0.019*	
C3B	1.01917 (14)	0.28581 (12)	0.37345 (11)	0.0181 (3)	
H3B1	1.0607	0.2080	0.3936	0.022*	
H3B2	0.9659	0.3077	0.4333	0.022*	
C4B	1.12643 (14)	0.38092 (12)	0.37728 (12)	0.0198 (3)	
H4B1	1.1803	0.3900	0.4588	0.024*	
H4B2	1.1851	0.3552	0.3231	0.024*	
C5B	1.06533 (13)	0.50073 (12)	0.34018 (12)	0.0171 (3)	
H5B1	1.1364	0.5597	0.3390	0.020*	
H5B2	1.0144	0.5303	0.3994	0.020*	
C6B	0.97415 (13)	0.49000 (11)	0.21687 (11)	0.0153 (3)	
H6B1	1.0276	0.4710	0.1565	0.018*	
H6B2	0.9318	0.5679	0.1988	0.018*	
C7B	0.78001 (12)	0.37651 (11)	0.08113 (11)	0.0137 (3)	
H7B	0.7038	0.3242	0.0880	0.016*	
C8B	0.84277 (13)	0.31733 (11)	−0.01577 (11)	0.0157 (3)	
C9B	0.74329 (14)	0.28622 (12)	−0.12940 (12)	0.0189 (3)	
H9B1	0.6784	0.2275	−0.1132	0.023*	
H9B2	0.7891	0.2492	−0.1892	0.023*	
C10B	0.67096 (13)	0.39844 (12)	−0.17934 (12)	0.0181 (3)	
H10C	0.7332	0.4512	−0.2084	0.022*	
H10D	0.5991	0.3749	−0.2474	0.022*	
C11B	0.61377 (13)	0.46594 (12)	−0.08390 (12)	0.0167 (3)	
H11C	0.5427	0.4173	−0.0628	0.020*	
H11D	0.5749	0.5415	−0.1153	0.020*	
C12B	0.72028 (13)	0.49357 (11)	0.02716 (12)	0.0155 (3)	
H12C	0.6817	0.5397	0.0868	0.019*	
H12D	0.7908	0.5430	0.0063	0.019*	
C13B	0.65969 (14)	0.32216 (12)	0.32900 (12)	0.0196 (3)	
H13C	0.6113	0.2890	0.2510	0.024*	
H13D	0.7167	0.2591	0.3687	0.024*	
C14B	0.56281 (15)	0.36372 (14)	0.40545 (14)	0.0266 (3)	
H14D	0.6116	0.3972	0.4820	0.040*	
H14E	0.5088	0.2957	0.4188	0.040*	
H14F	0.5057	0.4250	0.3647	0.040*	

### Atomic displacement parameters (Å^2^)

**Table:**

	*U*^11^	*U*^22^	*U*^33^	*U*^12^	*U*^13^	*U*^23^
S1A	0.01464 (16)	0.01284 (16)	0.01680 (16)	0.00131 (12)	0.00018 (12)	−0.00173 (12)
O1A	0.0168 (5)	0.0218 (5)	0.0189 (5)	−0.0030 (4)	0.0018 (4)	0.0022 (4)
C1A	0.0126 (6)	0.0127 (6)	0.0129 (6)	0.0011 (5)	0.0008 (5)	0.0005 (5)
C2A	0.0170 (6)	0.0128 (6)	0.0156 (6)	0.0005 (5)	0.0029 (5)	0.0005 (5)
C3A	0.0226 (7)	0.0164 (7)	0.0162 (6)	−0.0002 (5)	0.0050 (5)	0.0020 (5)
C4A	0.0203 (7)	0.0219 (7)	0.0175 (7)	0.0018 (5)	0.0070 (5)	0.0008 (5)
C5A	0.0163 (6)	0.0177 (7)	0.0172 (6)	0.0035 (5)	0.0029 (5)	−0.0024 (5)
C6A	0.0156 (6)	0.0135 (6)	0.0168 (6)	0.0017 (5)	0.0026 (5)	0.0008 (5)
C7A	0.0126 (6)	0.0119 (6)	0.0149 (6)	0.0008 (5)	0.0024 (5)	0.0004 (5)
C8A	0.0189 (7)	0.0112 (6)	0.0153 (6)	−0.0001 (5)	0.0014 (5)	0.0028 (5)
C9A	0.0212 (7)	0.0196 (7)	0.0148 (6)	−0.0035 (5)	0.0023 (5)	−0.0020 (5)
C10A	0.0183 (7)	0.0191 (7)	0.0155 (6)	0.0004 (5)	0.0033 (5)	0.0024 (5)
C11A	0.0162 (6)	0.0155 (6)	0.0177 (6)	−0.0002 (5)	0.0052 (5)	0.0021 (5)
C12A	0.0164 (6)	0.0130 (6)	0.0174 (6)	−0.0002 (5)	0.0040 (5)	0.0008 (5)
C13A	0.0161 (6)	0.0156 (6)	0.0171 (6)	0.0033 (5)	0.0012 (5)	0.0000 (5)
C14A	0.0169 (7)	0.0240 (7)	0.0180 (7)	0.0032 (5)	0.0018 (5)	−0.0006 (5)
S1B	0.02009 (17)	0.01311 (16)	0.01656 (17)	−0.00010 (12)	0.00800 (13)	−0.00127 (12)
O1B	0.0182 (5)	0.0221 (5)	0.0192 (5)	0.0057 (4)	0.0060 (4)	0.0018 (4)
C1B	0.0152 (6)	0.0118 (6)	0.0137 (6)	0.0008 (5)	0.0047 (5)	−0.0004 (5)
C2B	0.0192 (6)	0.0124 (6)	0.0151 (6)	0.0011 (5)	0.0038 (5)	0.0001 (5)
C3B	0.0235 (7)	0.0156 (7)	0.0143 (6)	0.0038 (5)	0.0018 (5)	0.0014 (5)
C4B	0.0202 (7)	0.0211 (7)	0.0168 (6)	0.0026 (5)	0.0011 (5)	−0.0012 (5)
C5B	0.0183 (7)	0.0156 (6)	0.0168 (6)	−0.0022 (5)	0.0031 (5)	−0.0021 (5)
C6B	0.0174 (6)	0.0128 (6)	0.0162 (6)	−0.0008 (5)	0.0046 (5)	0.0006 (5)
C7B	0.0151 (6)	0.0118 (6)	0.0147 (6)	0.0006 (5)	0.0040 (5)	0.0002 (5)
C8B	0.0192 (7)	0.0118 (6)	0.0168 (6)	0.0032 (5)	0.0054 (5)	0.0017 (5)
C9B	0.0210 (7)	0.0203 (7)	0.0151 (6)	0.0044 (5)	0.0037 (5)	−0.0028 (5)
C10B	0.0187 (7)	0.0207 (7)	0.0145 (6)	0.0010 (5)	0.0023 (5)	0.0022 (5)
C11B	0.0158 (6)	0.0154 (6)	0.0184 (6)	0.0018 (5)	0.0018 (5)	0.0025 (5)
C12B	0.0165 (6)	0.0122 (6)	0.0178 (6)	0.0020 (5)	0.0034 (5)	0.0010 (5)
C13B	0.0213 (7)	0.0183 (7)	0.0210 (7)	−0.0022 (5)	0.0083 (6)	0.0015 (5)
C14B	0.0274 (8)	0.0280 (8)	0.0282 (8)	−0.0014 (6)	0.0146 (6)	0.0004 (6)

### Geometric parameters (Å, °)

**Table:**

S1A—C13A	1.8130 (13)	S1B—C13B	1.8113 (14)
S1A—C1A	1.8577 (13)	S1B—C1B	1.8571 (13)
O1A—C8A	1.2189 (16)	O1B—C8B	1.2186 (16)
C1A—C2A	1.5399 (17)	C1B—C2B	1.5392 (17)
C1A—C6A	1.5440 (17)	C1B—C6B	1.5444 (17)
C1A—C7A	1.5504 (17)	C1B—C7B	1.5490 (17)
C2A—C3A	1.5281 (17)	C2B—C3B	1.5295 (18)
C2A—H2A1	0.9900	C2B—H2B1	0.9900
C2A—H2A2	0.9900	C2B—H2B2	0.9900
C3A—C4A	1.5324 (19)	C3B—C4B	1.5303 (19)
C3A—H3A1	0.9900	C3B—H3B1	0.9900
C3A—H3A2	0.9900	C3B—H3B2	0.9900
C4A—C5A	1.5293 (19)	C4B—C5B	1.5279 (19)
C4A—H4A1	0.9900	C4B—H4B1	0.9900
C4A—H4A2	0.9900	C4B—H4B2	0.9900
C5A—C6A	1.5323 (18)	C5B—C6B	1.5354 (18)
C5A—H5A1	0.9900	C5B—H5B1	0.9900
C5A—H5A2	0.9900	C5B—H5B2	0.9900
C6A—H6A1	0.9900	C6B—H6B1	0.9900
C6A—H6A2	0.9900	C6B—H6B2	0.9900
C7A—C8A	1.5265 (17)	C7B—C8B	1.5290 (17)
C7A—C12A	1.5507 (17)	C7B—C12B	1.5535 (17)
C7A—H7A	1.0000	C7B—H7B	1.0000
C8A—C9A	1.5179 (18)	C8B—C9B	1.5168 (18)
C9A—C10A	1.5387 (18)	C9B—C10B	1.5398 (18)
C9A—H9A1	0.9900	C9B—H9B1	0.9900
C9A—H9A2	0.9900	C9B—H9B2	0.9900
C10A—C11A	1.5221 (18)	C10B—C11B	1.5242 (18)
C10A—H10A	0.9900	C10B—H10C	0.9900
C10A—H10B	0.9900	C10B—H10D	0.9900
C11A—C12A	1.5262 (17)	C11B—C12B	1.5278 (18)
C11A—H11A	0.9900	C11B—H11C	0.9900
C11A—H11B	0.9900	C11B—H11D	0.9900
C12A—H12A	0.9900	C12B—H12C	0.9900
C12A—H12B	0.9900	C12B—H12D	0.9900
C13A—C14A	1.5256 (19)	C13B—C14B	1.5249 (19)
C13A—H13A	0.9900	C13B—H13C	0.9900
C13A—H13B	0.9900	C13B—H13D	0.9900
C14A—H14A	0.9800	C14B—H14D	0.9800
C14A—H14B	0.9800	C14B—H14E	0.9800
C14A—H14C	0.9800	C14B—H14F	0.9800
			
C13A—S1A—C1A	104.36 (6)	C13B—S1B—C1B	104.78 (6)
C2A—C1A—C6A	109.67 (10)	C2B—C1B—C6B	109.61 (10)
C2A—C1A—C7A	111.20 (10)	C2B—C1B—C7B	111.00 (10)
C6A—C1A—C7A	112.51 (10)	C6B—C1B—C7B	112.67 (10)
C2A—C1A—S1A	110.62 (8)	C2B—C1B—S1B	110.81 (8)
C6A—C1A—S1A	104.83 (8)	C6B—C1B—S1B	104.68 (8)
C7A—C1A—S1A	107.81 (8)	C7B—C1B—S1B	107.89 (8)
C3A—C2A—C1A	113.55 (10)	C3B—C2B—C1B	113.36 (10)
C3A—C2A—H2A1	108.9	C3B—C2B—H2B1	108.9
C1A—C2A—H2A1	108.9	C1B—C2B—H2B1	108.9
C3A—C2A—H2A2	108.9	C3B—C2B—H2B2	108.9
C1A—C2A—H2A2	108.9	C1B—C2B—H2B2	108.9
H2A1—C2A—H2A2	107.7	H2B1—C2B—H2B2	107.7
C2A—C3A—C4A	110.79 (11)	C2B—C3B—C4B	110.77 (11)
C2A—C3A—H3A1	109.5	C2B—C3B—H3B1	109.5
C4A—C3A—H3A1	109.5	C4B—C3B—H3B1	109.5
C2A—C3A—H3A2	109.5	C2B—C3B—H3B2	109.5
C4A—C3A—H3A2	109.5	C4B—C3B—H3B2	109.5
H3A1—C3A—H3A2	108.1	H3B1—C3B—H3B2	108.1
C5A—C4A—C3A	110.49 (11)	C5B—C4B—C3B	110.68 (11)
C5A—C4A—H4A1	109.6	C5B—C4B—H4B1	109.5
C3A—C4A—H4A1	109.6	C3B—C4B—H4B1	109.5
C5A—C4A—H4A2	109.6	C5B—C4B—H4B2	109.5
C3A—C4A—H4A2	109.6	C3B—C4B—H4B2	109.5
H4A1—C4A—H4A2	108.1	H4B1—C4B—H4B2	108.1
C4A—C5A—C6A	111.50 (11)	C4B—C5B—C6B	111.64 (11)
C4A—C5A—H5A1	109.3	C4B—C5B—H5B1	109.3
C6A—C5A—H5A1	109.3	C6B—C5B—H5B1	109.3
C4A—C5A—H5A2	109.3	C4B—C5B—H5B2	109.3
C6A—C5A—H5A2	109.3	C6B—C5B—H5B2	109.3
H5A1—C5A—H5A2	108.0	H5B1—C5B—H5B2	108.0
C5A—C6A—C1A	113.11 (10)	C5B—C6B—C1B	112.80 (10)
C5A—C6A—H6A1	109.0	C5B—C6B—H6B1	109.0
C1A—C6A—H6A1	109.0	C1B—C6B—H6B1	109.0
C5A—C6A—H6A2	109.0	C5B—C6B—H6B2	109.0
C1A—C6A—H6A2	109.0	C1B—C6B—H6B2	109.0
H6A1—C6A—H6A2	107.8	H6B1—C6B—H6B2	107.8
C8A—C7A—C1A	118.12 (10)	C8B—C7B—C1B	117.75 (10)
C8A—C7A—C12A	104.50 (10)	C8B—C7B—C12B	104.56 (10)
C1A—C7A—C12A	114.47 (10)	C1B—C7B—C12B	114.68 (10)
C8A—C7A—H7A	106.3	C8B—C7B—H7B	106.4
C1A—C7A—H7A	106.3	C1B—C7B—H7B	106.4
C12A—C7A—H7A	106.3	C12B—C7B—H7B	106.4
O1A—C8A—C9A	121.75 (12)	O1B—C8B—C9B	121.68 (12)
O1A—C8A—C7A	125.31 (12)	O1B—C8B—C7B	125.40 (12)
C9A—C8A—C7A	112.83 (11)	C9B—C8B—C7B	112.78 (11)
C8A—C9A—C10A	110.85 (11)	C8B—C9B—C10B	110.92 (11)
C8A—C9A—H9A1	109.5	C8B—C9B—H9B1	109.5
C10A—C9A—H9A1	109.5	C10B—C9B—H9B1	109.5
C8A—C9A—H9A2	109.5	C8B—C9B—H9B2	109.5
C10A—C9A—H9A2	109.5	C10B—C9B—H9B2	109.5
H9A1—C9A—H9A2	108.1	H9B1—C9B—H9B2	108.0
C11A—C10A—C9A	110.79 (11)	C11B—C10B—C9B	110.61 (11)
C11A—C10A—H10A	109.5	C11B—C10B—H10C	109.5
C9A—C10A—H10A	109.5	C9B—C10B—H10C	109.5
C11A—C10A—H10B	109.5	C11B—C10B—H10D	109.5
C9A—C10A—H10B	109.5	C9B—C10B—H10D	109.5
H10A—C10A—H10B	108.1	H10C—C10B—H10D	108.1
C10A—C11A—C12A	110.75 (11)	C10B—C11B—C12B	110.84 (11)
C10A—C11A—H11A	109.5	C10B—C11B—H11C	109.5
C12A—C11A—H11A	109.5	C12B—C11B—H11C	109.5
C10A—C11A—H11B	109.5	C10B—C11B—H11D	109.5
C12A—C11A—H11B	109.5	C12B—C11B—H11D	109.5
H11A—C11A—H11B	108.1	H11C—C11B—H11D	108.1
C11A—C12A—C7A	110.79 (10)	C11B—C12B—C7B	110.76 (10)
C11A—C12A—H12A	109.5	C11B—C12B—H12C	109.5
C7A—C12A—H12A	109.5	C7B—C12B—H12C	109.5
C11A—C12A—H12B	109.5	C11B—C12B—H12D	109.5
C7A—C12A—H12B	109.5	C7B—C12B—H12D	109.5
H12A—C12A—H12B	108.1	H12C—C12B—H12D	108.1
C14A—C13A—S1A	107.95 (9)	C14B—C13B—S1B	107.97 (10)
C14A—C13A—H13A	110.1	C14B—C13B—H13C	110.1
S1A—C13A—H13A	110.1	S1B—C13B—H13C	110.1
C14A—C13A—H13B	110.1	C14B—C13B—H13D	110.1
S1A—C13A—H13B	110.1	S1B—C13B—H13D	110.1
H13A—C13A—H13B	108.4	H13C—C13B—H13D	108.4
C13A—C14A—H14A	109.5	C13B—C14B—H14D	109.5
C13A—C14A—H14B	109.5	C13B—C14B—H14E	109.5
H14A—C14A—H14B	109.5	H14D—C14B—H14E	109.5
C13A—C14A—H14C	109.5	C13B—C14B—H14F	109.5
H14A—C14A—H14C	109.5	H14D—C14B—H14F	109.5
H14B—C14A—H14C	109.5	H14E—C14B—H14F	109.5
			
C13A—S1A—C1A—C2A	49.82 (10)	C13B—S1B—C1B—C2B	−47.41 (10)
C13A—S1A—C1A—C6A	167.97 (8)	C13B—S1B—C1B—C6B	−165.49 (8)
C13A—S1A—C1A—C7A	−71.97 (9)	C13B—S1B—C1B—C7B	74.30 (10)
C6A—C1A—C2A—C3A	−52.42 (14)	C6B—C1B—C2B—C3B	53.18 (14)
C7A—C1A—C2A—C3A	−177.51 (11)	C7B—C1B—C2B—C3B	178.28 (11)
S1A—C1A—C2A—C3A	62.73 (12)	S1B—C1B—C2B—C3B	−61.86 (13)
C1A—C2A—C3A—C4A	55.83 (15)	C1B—C2B—C3B—C4B	−56.10 (14)
C2A—C3A—C4A—C5A	−56.44 (15)	C2B—C3B—C4B—C5B	56.19 (14)
C3A—C4A—C5A—C6A	56.25 (14)	C3B—C4B—C5B—C6B	−55.90 (15)
C4A—C5A—C6A—C1A	−54.81 (15)	C4B—C5B—C6B—C1B	54.72 (15)
C2A—C1A—C6A—C5A	51.57 (14)	C2B—C1B—C6B—C5B	−52.08 (14)
C7A—C1A—C6A—C5A	175.90 (10)	C7B—C1B—C6B—C5B	−176.21 (10)
S1A—C1A—C6A—C5A	−67.21 (12)	S1B—C1B—C6B—C5B	66.82 (12)
C2A—C1A—C7A—C8A	51.95 (15)	C2B—C1B—C7B—C8B	−51.91 (14)
C6A—C1A—C7A—C8A	−71.53 (14)	C6B—C1B—C7B—C8B	71.45 (14)
S1A—C1A—C7A—C8A	173.37 (9)	S1B—C1B—C7B—C8B	−173.49 (9)
C2A—C1A—C7A—C12A	175.66 (10)	C2B—C1B—C7B—C12B	−175.58 (10)
C6A—C1A—C7A—C12A	52.18 (14)	C6B—C1B—C7B—C12B	−52.22 (14)
S1A—C1A—C7A—C12A	−62.92 (12)	S1B—C1B—C7B—C12B	62.84 (12)
C1A—C7A—C8A—O1A	14.12 (19)	C1B—C7B—C8B—O1B	−14.47 (19)
C12A—C7A—C8A—O1A	−114.43 (14)	C12B—C7B—C8B—O1B	114.15 (14)
C1A—C7A—C8A—C9A	−169.72 (11)	C1B—C7B—C8B—C9B	169.73 (11)
C12A—C7A—C8A—C9A	61.73 (13)	C12B—C7B—C8B—C9B	−61.65 (13)
O1A—C8A—C9A—C10A	118.09 (13)	O1B—C8B—C9B—C10B	−117.53 (14)
C7A—C8A—C9A—C10A	−58.22 (14)	C7B—C8B—C9B—C10B	58.46 (14)
C8A—C9A—C10A—C11A	51.80 (15)	C8B—C9B—C10B—C11B	−52.11 (15)
C9A—C10A—C11A—C12A	−53.81 (14)	C9B—C10B—C11B—C12B	53.95 (14)
C10A—C11A—C12A—C7A	61.17 (14)	C10B—C11B—C12B—C7B	−61.06 (14)
C8A—C7A—C12A—C11A	−62.58 (13)	C8B—C7B—C12B—C11B	62.32 (13)
C1A—C7A—C12A—C11A	166.68 (10)	C1B—C7B—C12B—C11B	−167.23 (10)
C1A—S1A—C13A—C14A	174.74 (9)	C1B—S1B—C13B—C14B	−176.72 (10)

## References

[bb1] Bach, R. D. & Klix, R. C. (1985). *J. Org. Chem.* **50**, 5440–5441.

[bb2] Ito, Y., Fujii, S., Nakatuska, M., Kawamoto, F. & Saegusa, T. (1979). *Org. Synth.* **59**, 113–121.

[bb3] Kumar, V. & Dev, S. (1983). *Tetrahedron Lett.* **24**, 1289–1292.

[bb4] Nonius (1998). *COLLECT* Nonius BV, Delft, The Netherlands.

[bb5] Otwinowski, Z. & Minor, W. (1997). *Methods in Enzymology*, Vol. 276, *Macromolecular Crystallography*, Part A, edited by C. W. Carter Jr & R. M. Sweet, pp. 307–326. New York: Academic Press.

[bb6] Reetz, M. T. & Giannis, A. (1981). *Synth. Commun.* **11**, 315–322.

[bb7] Sheldrick, G. M. (2008). *Acta Cryst.* A**64**, 112–122. 10.1107/S0108767307043930 18156677

[bb8] Trost, B. M., Salzmann, T. N. & Hirori, K. (1976). *J. Am. Chem. Soc.* **98**, 4887–4902.

